# The Heating and Lighting of Hospital Wards

**Published:** 1909-01-09

**Authors:** 


### HOSPITAL ADMINISTRATION.

### CONSTRUCTION AND ECONOMICS.

### THE HEATING AND LIGHTING OF HOSPITAL WARDS.

#### I.—METHODS OF HEATING.

The oldest method of heating wards and the one still in use in a large number of cases is by means of the open fireplace. There is a great prejudice among Englishmen generally in favour of the open fireplace: it is so cheerful. But it is very wasteful and very inefficient; the major portion of the heat given out by the burning coal passes up the chimney, and the radiant heat given off by the glowing coals does not heat the air. If a room is well warmed by coal fires it is only when and because furniture arrests and absorbs the heat rays, sets up convection currents, and reflects the heat rays.

The next method, and one that has found great favour, is the air-heating stove. This was designed and first introduced by the late Dr. Pridgin Teale. It is now made by a large number of firms, and for almost every position—the end of the ward, or, if place can be found for it, the middle of one of the long walls of the ward, and the middle of the floor. It is also adapted for smaller rooms, such as nurses' sitting-rooms, bedrooms, etc. The special feature of the stove is the provision of a space behind the flue of the fireplace, over which air from outside the building is allowed to pass; the air is warmed by contact with the heated flue, and enters the ward at about the level of the usual chimney breast, sometimes with a slight inclination upward, at a moderately high temperature. This form of fireplace combines the cheerful effect of the ordinary open fireplace with a provision of warmed air circulating through the ward. On Dr. Teale's original fireplace the arrangement of sides and back also played an important part in reflecting heat rays outwards. Some of the wards of modern hospitals are very efficiently warmed by large fireplaces on these lines, one at one end of the ward and one or two double fireplaces standing back to back and enclosed in either ornamental ironwork or tiles in the middle of the floor of the ward. This form of stove suffers from one disadvantage, common to every form of coal-burning stove, namely coal has to be brought to each ward and must be stored in the ward, in sufficient quantities to enable the nurses to keep the fires going. All open fireplaces also, burning coal, have the disadvantage as compared with other methods of heating that they create dust, and both the presence of dust and the necessity for feeding the fire give additional work to the nurses.

Gas is used very largely in private houses for heating, but the writer is not aware of its being used to any large extent in hospitals. The ordinary gas fire, in which some form of Bunsen burner consumes gas and air and maintains a refractory substance at a high temperature suffers from the same disadvantage as the open coal fireplace. The major portion of the heat liberated by the burning gas passes up the flue and into the chimney. In addition, gas fires suffer from two somewhat serious troubles, though they are often very convenient in small sick-rooms. The heat in the immediate neighbourhood of the gas fire dries the air, and unless a pan of water is placed near, the result upon the atmosphere in the neighbourhood of the fire is unpleasant. It is also sometimes very difficult to ensure that there shall be no escape of the unburned gas into the room, though with modern appliances, careful fitting, and with properly arranged flues leading to the chimney, the danger of this should not be serious. Gas engineers have also borrowed the idea of the air-heating stove, and gas fires are fitted with the space behind the flue, mentioned in connection with the air-heating coal stove, air being passed through it and delivered to the room to be warmed at a comparatively high temperature. It is claimed by one of the leading exponents of gas practice that an economy of 15 per cent. in the gas is obtained by this means. The use of gas has the great advantage that there is no fuel to be taken to the ward, and no attention is required in the matter of stoking.

The requirements of modern hospitals, however, in the matter of hot water, their greatly increased size, and greatly increased comfort above those of bygone days, added to the necessity of the study of economy, have gradually led to the development of heating systems, in which the fuel generating the heat is consumed in some convenient spot in the basement or elsewhere, and is employed either to heat water in boilers arranged for the purpose, or to generate steam. The arrangement of the two systems will be described in another article. Meanwhile it may be mentioned that the heated water, or the steam, is carried from the basement to every floor and to every part where heat is required, and is there employed in heating groups of metal pipes known as radiators. The great advantage of the radiator, quite apart from the matter of economy is—that any number can be placed about a ward and in any position where heat may be required. Further, with a properly arranged system of radiators, whether heated by hot water or by steam, it should be possible to regulate the temperature of the surface of the radiator, and therefore the rate at which it is delivering heat to the surrounding atmosphere, and thence the temperature of the ward or any portion of the ward. The radiators are also sometimes combined with a fresh air supply, air being taken from the outside atmosphere, and passed over the surface of the radiator, before it enters the ward, the result being an assistance to the ventilation as well as the warming of the atmosphere of the ward.

As will be explained in the next article also, electricity is now employed for delivering heat to radiators, most of them differing in form from those supplied by hot water or steam, but in the latest form placed on the market the hot water and steam radiator have been closely copied.

